# Cervical Intervertebral Disk to Vertebral Body Ratios of Different Dog Breeds Based on Sagittal Magnetic Resonance Imaging

**DOI:** 10.3389/fvets.2018.00248

**Published:** 2018-10-05

**Authors:** Pia Düver, Christina Precht, Geoffrey Fosgate, Franck Forterre, Bianca Hettlich

**Affiliations:** ^1^Division of Small Animal Surgery, Department of Clinical Veterinary Medicine, Vetsuisse Faculty, University of Bern, Bern, Switzerland; ^2^Division of Clinical Radiology, Department of Clinical Veterinary Medicine, Vetsuisse Faculty, University of Bern, Bern, Switzerland; ^3^Department of Production Animal Studies, Faculty of Veterinary Science, University of Pretoria, Onderstepoort, South Africa

**Keywords:** canine, cervical vertebral column, morphometry, intervertebral disk, magnetic resonance imaging, area, length, ratio

## Abstract

**Objective:** To establish sagittal area and length reference values and ratios between apparently normal canine cervical vertebrae and intervertebral disks using magnetic resonance imaging.

**Sample:** Retrospective evaluation of cervical vertebral column magnetic resonance imaging studies of 44 dogs representing 5 different breeds (Labrador Retriever, *n* = 10; French Bulldog, *n* = 10; Great Dane, *n* = 9; Chihuahua, *n* = 10; Dachshund, *n* = 5).

**Procedures:** Mid-sagittal measurements of vertebral body and disk areas were obtained from C3 through C7 vertebrae and C2/C3 through C6/C7 intervertebral disks. Disk to vertebra area ratios were calculated and compared among dog breeds. Additionally, sagittal vertebral body and disk length measurements were obtained and disk to vertebra length ratios calculated. Inter and intra observer variability was assessed.

**Results:** There were significant differences for disk to vertebral body area and length ratios between evaluated dog breeds and cervical vertebral locations (*p* < 0.001). Mean area ratio of Chihuahuas was significantly larger than all other breeds, while results from Dachshunds were only significantly different than Chihuahuas and Labrador Retrievers. Mean area ratios were statistically different between the cranial and caudal cervical vertebral locations. Regarding length ratios, results from Chihuahuas were significantly different than all breeds except Dachshunds. Mean length ratios were statistically different between all cervical locations, except C2/C3 compared to C3/C4. Intra- and interobserver variability was very good to excellent.

**Conclusion and Clinical Relevance:** There are significant differences in area and length ratios between dog breeds. Differences also exist in area and length ratios between the cranial and caudal cervical vertebral column. These differences may play a role in the development of vertebral column diseases including intervertebral disk disease.

## Introduction

Morphometry, a quantitative analysis of the size and shape of a form, has been used in human and animal studies to identify relationships between specific anatomic structures and diseases (1–6). The canine vertebral column has been morphometrically evaluated using anatomic specimens (macerated vertebrae) and imaging modalities, such as radiography, computed tomography (CT) and magnetic resonance imaging (MRI) (7–12).

Common disorders of the canine cervical vertebral column include intervertebral disk diseases (IVDD) and cervical spondylomyelpathy (CSM) (13–17).

IVDD often affects chondrodystrophic dogs and is characterized by degeneration of the intervertebral disk with possible spinal cord compression by disk extrusion or protrusion (14, 15). Approximately 15% of IVDD occurs in the cervical vertebral column in dogs (18). CSM is a disease mainly of large and giant breed dogs causing cervical myelopathy due to progressive spinal cord compression (17). CSM has been divided into two distinctly different types: osseous-associated and disk-associated (OA-CSM and DA-CSM, respectively). Both of these diseases have been evaluated morphometrically using various imaging modalities (13, 19–24).

MRI analysis of cervical vertebral columns of Great Danes demonstrated morphometric differences between OA-CSM affected and non-affected dogs, including smaller intervertebral disk widths, smaller vertebral canal areas and smaller spinal cord areas (21). Regarding DA-CSM, morphometric MRI comparison of affected and non-affected Doberman Pinchers showed smaller spinal cord area, smaller vertebral canal height and more square shaped vertebral bodies compared to their normal counterparts (19, 20). Disk width was evaluated as a stand-alone morphometric MRI measurement in dogs with and without DA-CSM and was found to be positively associated with age but not associated with the clinical status of the dog (22). In another morphometric study using MRI, articular process conformation was compared between Doberman Pinchers and Great Danes. Dobermans were found to have more concave caudal articular surfaces in caudal cervical locations, a morphometric difference which was proposed to explain the high incidence of DA-CSM in Dobermans (25).

There appears to only be one morphometric study evaluating area measurements of vertebral bodies in animals (26). In this study, vertebral canal and vertebral body area (among various other measurements) was calculated using CT in a rat model for human disease. We are unaware of studies reporting on the use of area measurements of disk and vertebral body of cervical vertebral columns in different dog breeds and different cervical vertebral locations. Data regarding these ratios may be useful to try to link conformation to the development of diseases, such as IVDD and CSM.

The purpose of this study was to determine disk to vertebral body ratios at different cervical vertebral locations and in different dog breeds using mid-sagittal MRI images. We hypothesized that (1) there would be a difference in area and length ratios based on location within the cervical vertebral column and (2) between different breeds of dogs.

## Materials and methods

The medical record system of the Vetsuisse Faculty, University of Bern, Switzerland, was searched for canine cervical vertebral column MRI studies performed between 2009 and 2016 through the Division of Clinical Radiology. Studies had to be obtained with proper patient positioning, be of good quality and be free of artifacts impairing evaluation of vertebral bodies and intervertebral disks (IVD). Dogs had to be mature based on physeal closure. Studies had to include the vertebral column from C2 to T1 and sagittal T2 weighted images of this area had to be available. Vertebrae C3 to C7 and intervertebral disks C2/C3 through C6/C7 were evaluated but only vertebrae and disks free of diseases, which would affect their imaging appearance and shape, were measured. Disks with advanced degeneration, protrusion or extrusion, or adjacent tissue changes that influenced disk appearance were excluded. Deformed, fractured or otherwise distorted vertebral bodies (i.e., due to neoplasia) and vertebrae with new bone formation, such as spondylosis deformans were also excluded. Intervertebral disks were evaluated using Pfirrmann grades, an MRI based grading system classifying intervertebral disks and stages of degeneration (27). Briefly, Pfirrmann Grade 1 IVD is considered normal and shows the nucleus pulposus as a bright white homogeneous structure. Nucleus pulposus and annulus fibrosus can be clearly distinguished and the IVD shows a normal size. Grade 2 describes a nonhomogeneous structure of the IVD with clear distinguished nucleus pulposus and annulus fibrosus and normal disk space. The higher the Pfirrmann grade, the more IVD degeneration is present, with a black disk, no distinction between annulus fibrosus and nucleus pulposus, a hypointense signal, and a collapsed disk space representing the highest grade (grade 5). Only IVDs with a Pfirrmann grade of 2 or less were included in this study.

### Measurements

Patient positioning was standardized with dogs positioned in dorsal recumbency with the head and neck extended and thoracic limbs pulled caudally. Images were obtained using a MRI with 1.0 Tesla field strength and fast spin-echo T2-weighted sequence in sagittal plane with repetition time 2,500–6,000 ms, echo time 100 ms, slice thickness 2.5–4.0 mm and gap 0.5 mm depending on the size of the patient (Philips Panorama HFO, Philips Medical Systems Nederland B.V., Best, Netherlands). All measurements were performed on midsagittal T2 weighted images of the vertebral column section evaluated. Midsagittal plane was identified by help of a concurrently displayed linked dorsal plane image using an imaging software program (IMPAX EE R20.Ink, AGFA HealthCare N.V., Belgium). Tools of the same program were used to outline vertebral bodies and IVDs and obtain area and length measurements.

#### Area measurements

**Figure 1:**
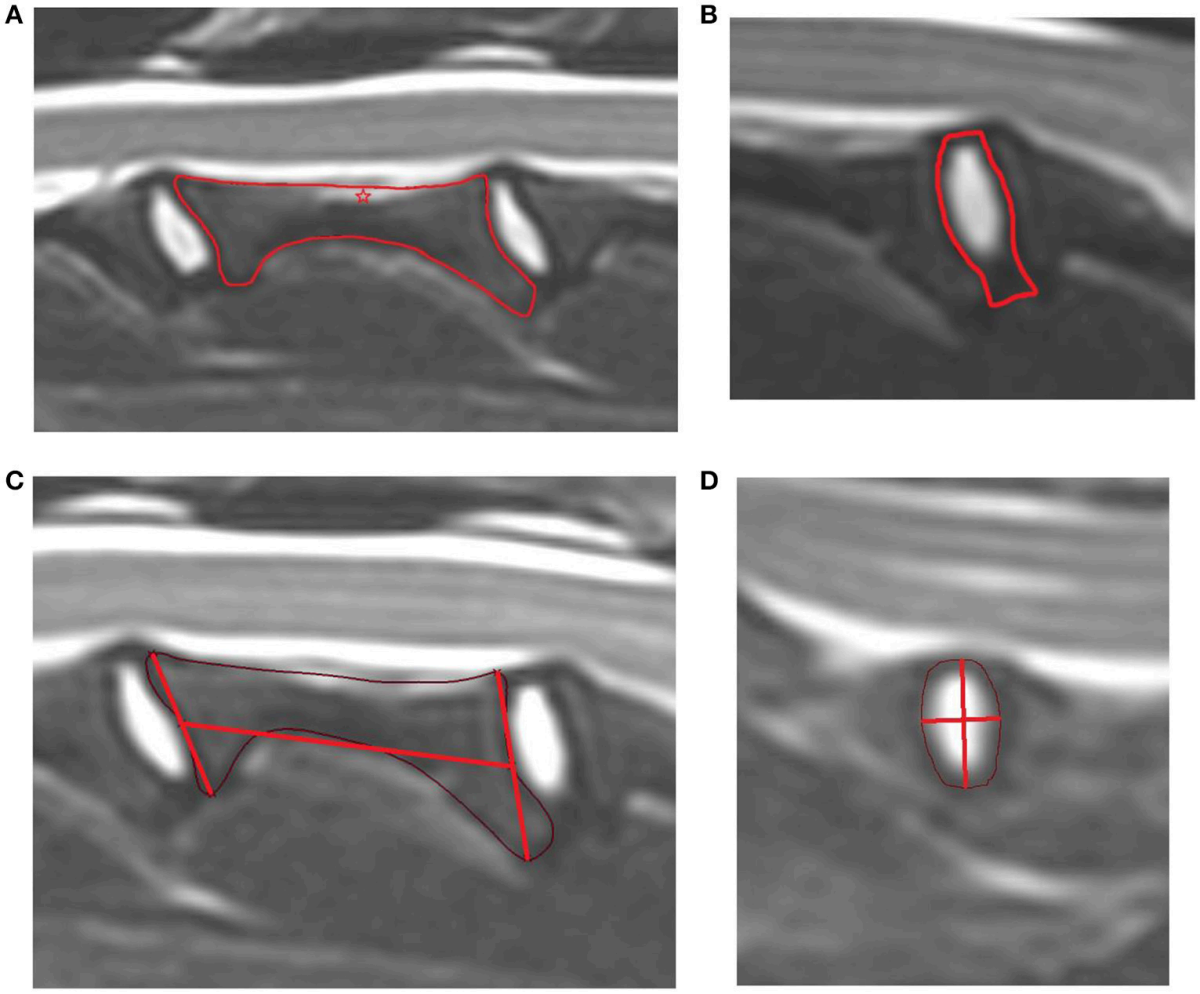
**(A)** Example of the area measurement of a cervical vertebral body (red line) in a Labrador retriever. The star indicates the area in the middle of the dorsal vertebral body where the cortex cannot be followed continuously and a line of best fit was drawn. **(B)** Example of the area measurement of a cervical intervertebral disk (red line) in a Labrador Retriever. **(C)** Example of the length measurement of a cervical vertebral body in a Labrador retriever. The area outline was performed (thin purple line), then lines were drawn along the cranial and caudal endplate from the furthest dorsal and ventral aspect of the area outline (vertical red lines). The midpoints of these lines were determined and a connecting line through the midpoints was used to measure the length of the vertebral body, reaching from the most cranial to caudal cortex. **(D)** Example of the length measurement of a cervical intervertebral disk in a Chihuahua. The area outline was performed (thin red line) and a vertical line drawn from the most dorsal to ventral aspect. At the middle distance of this line, a second horizontal line was drawn at a right angle from cranial to caudal area outline. This horizontal line was used for length measurement of the disk.

Vertebral bodies (Figure 1A): A line was drawn along the outer vertebral cortex of each vertebral body. In areas of poorly defined cortical bone continuity, such as the mid-portion of the dorsal cortex of the vertebral body (ventral aspect of vertebral canal), a “best-fit” line was drawn to connect the two points of visible cortex. Due to the inability to distinguish between the hypointense cortical bone and annulus fibrosus along the vertebral endplates, the middle distance of the hypointense area between hyperintense medullary bone and nucleus pulposus was chosen as the border between cortical bone and annulus fibrosus.

Intervertebral disk (Figure 1B): A line was drawn around the outer limit of the annulus fibrosus dorsally and ventrally. Along the vertebral endplates, the previously described middle distance line was used to outline the remainder of the IVD.

The area ratio was calculated between the IVD and the vertebral body immediately caudal using the following equation:

$$area\;ratio=\frac{disk\;area}{vertrebra\;area}$$

#### Length measurement

Vertebral bodies (Figure 1C): The length of the vertebral body was measured from the midpoint of the cranial to the midpoint of the caudal vertebral endplate. To define these endplate midpoints, a line was drawn from the most prominent dorsal and to ventral bone protuberances associated with each of the cranial and caudal endplate. The midpoint of the line was determined. A connecting line through the midpoint of each of these lines was used to measure the length of the vertebral body, reaching from the most cranial to caudal cortex.

Intervertebral disk (Figure 1D): A height measurement line was drawn from the most dorsal to the most ventral aspect of the IVD. At the midpoint of this line, a perpendicular second line was drawn reaching from the cranial to caudal border of the IVD. This line represented the length of the IVD. The length ratio was calculated using the following equation:

$$length\;ratio=\;\frac{disk\;length}{vertrebra\;length}$$

### Intra- and interobserver variability

Measurements of all MRI studies were performed by one person using the same computer station and screen settings. To assess intraobserver variability, the same person repeated area and length measurements of 2 randomly selected MRI studies from each breed (except Dachshund) without access to previous measurement data. The timing between the first and the second measurement averaged 5 weeks.

To assess interobserver variability, these same 8 MRI studies used for intraobserver variability were measured by two other observers (board certified surgeon and a board certified radiologist). These observers were blinded to all previously obtained measurement data.

### Statistical analysis

Disk ratios were assessed for normality by calculating descriptive statistics, plotting histograms, and performing the Anderson-Darling test in commercial software (MINITAB Statistical Software, Release 13.32, Minitab Inc, State College, Pennsylvania, USA; NCSS 10 Statistical Software, Version 10.0.12, East Kaysville, Utah, USA). Disk ratios were described using the median and range due to the small sample size and an apparent violation of the normality assumption. Correlation between area and length ratios was assessed using Spearman's rho. The coefficient of variation was calculated to determine intra and inter observer variation among repeated measurements. The coefficient of variation is calculated as the standard deviation divided by the mean of repeated ratio measurements. Using the coefficient of variation, 0–5% was defined as “excellent,” 5–10% as “very good,” 10–15% as “good,” and 15–20% as “acceptable.” Natural logarithm transformed disk ratio data were analyzed using a mixed-effects linear regression model to determine the effects of breed and disk space. A random effect was included in the model for dog to account for the repeated measurements and fixed effect terms were included for breeds and disk space. Independent models were fit for area and length ratios and *post-hoc* pairwise comparisons were adjusted using Bonferroni correction of *p*-values. Mixed-effects models were implemented in commercially available software (IBM SPSS Statistics Version 23, International Business Machines Corp., Armonk, NY, USA) and significance was set as *p* < 0.05.

## Results

Between 2009 and 2016, 362 canine cervical vertebral column MRIs were performed at the Vetsuisse Faculty, University of Bern. Of the most common breeds represented, there were 50 cervical MRIs of French Bulldogs, 19 of Labrador Retrievers, 17 of Chihuahuas, 14 of Great Danes and 8 of Dachshunds. Studies of cervical MRIs of 44 dogs were ultimately included (Table 1) and represented five different dog breeds: Labrador retriever (LR, *n* = 10), Great Dane (GD, *n* = 9), French bulldog (FB, *n* = 10), Chihuahua (CH, *n* = 10), and Dachshund (DH, *n* = 5). Of these dogs, there were 21 females (15 spayed, 6 intact) and 23 males (15 intact, 8 neutered). The most common pathologies diagnosed were IVDD, OA-CSM, and intracranial disease (Table 2). In 3 MRI studies, no causative pathology was identified.

**Table 1:** Weight and age of 44 dogs including Labrador Retriever (LR, *n* = 10), Great Dane (GD, *n* = 9), French Bulldog (FB, *n* = 10), Chihuahua (CH, *n* = 10), and Dachshund (DH, *n* = 5).

	**Weight (kg)**					**Age (months)**		
**Breed**	**Mean**	**SD**	**Min**.	**Max**.	**Mean**	**SD**	**Min**.	**Max**.
LR	29.7	7.5	21.0	40.0	91.3	37.4	29	149
GD	69.8	9.0	60.0	85.0	40.8	20.9	15	75
FB	12.8	2.0	8.1	16.0	62.2	17.5	35	80
CH	2.6	1.1	1.5	5.3	51.3	26.9	20	90
DH	7.7	1.8	5.1	9.9	97.0	22.5	74	130

*SD, standard deviation*.

**Table 2:** Pathologies compatible with presenting clinical signs diagnosed on MRI.

		**Diseases affecting the region of interest of the study**			**Diseases of other locations**					
	**Normal**	**IVDD**	**CSM**	**Neoplasia**	**Trauma**	**Peripheral neuropathy**	**Arachnoid cyst**	**Syrinx**	**Intra-cranial**	**Atlantoaxial instability**
	***n* = 3**	***n* = 19**	***n* = 8**	***n* = 1**	***n* = 1**	***n* = 1**	***n* = 1**	***n* = 1**	***n* = 7**	***n* = 2**
LR *n* = 10	1	5	0	1	1	1	0	0	1	0
GD *n* = 9	1	0	8	0	0	0	0	0	0	0
FB *n* = 10	1	8	0	0	0	0	0	1	0	0
CH *n* = 10	0	2	0	0	0	0	1	0	5	2
DH *n* = 5	0	4	0	0	0	0	0	0	1	0

### Comparison of breeds

There were significant differences for disk to vertebral body area and length ratios between evaluated dog breeds and cervical vertebral locations (Table 3). The mean area ratio of Chihuahuas was significantly greater than all other evaluated breeds (Labrador retrievers, Great Danes, French bulldogs, and Dachshunds). Dachshunds also had significantly greater mean area ratios compared to Labrador retrievers. The mean length ratios of Chihuahuas were significantly greater than all other breeds except Dachshunds.

**Table 3:** Descriptive statistics and comparison of disk ratios based on breed and cervical disk based on a linear mixed-effects model.

**Measure**	**Variable**	**Level**	**Disk ratio**		***P*-value**
			**Mean**	**Median (IQR)**	
Area ratio	Breed				< 0.001
		LR	0.222^a^	0.211 (0.192, 0.250)	
		GD	0.227^a, b^	0.224 (0.201, 0.249)	
		FB	0.239^a, b^	0.252 (0.230, 0.282)	
		CH	0.349^c^	0.341 (0.313, 0.395)	
		DH	0.280^b^	0.287 (0.254, 0.310)	
	Location				< 0.001
		C2/C3	0.240^a^	0.226 (0.200, 0.263)	
		C3/4	0.249^a^	0.240 (0.190, 0.307)	
		C4/5	0.273^b^	0.262 (0.206, 0.324)	
		C5/6	0.274^b^	0.260 (0.228, 0.308)	
		C6/7	0.288^b^	0.283 (0.240, 0.343)	
Length ratio	Breed				< 0.001
		LR	0.216^a^	0.218 (0.182, 0.239)	
		GD	0.228^a, b^	0.222 (0.189, 0.260)	
		FB	0.214^a^	0.213 (0.175, 0.242)	
		CH	0.315^c^	0.308 (0.278, 0.358)	
		DH	0.279^b, c^	0.291 (0.228, 0.336)	
	Location				< 0.001
		C2/C3	0.206^a^	0.195 (0.169, 0.229)	
		C3/4	0.222^a^	0.214 (0.175, 0.247)	
		C4/5	0.245^b^	0.233 (0.203, 0.293)	
		C5/6	0.273^c^	0.258 (0.242, 0.319)	
		C6/7	0.294^d^	0.282 (0.259, 0.329)	

*IQR, interquartile range. The small letters a–d represent statistical differences—same letters indicate no difference, different letters indicate statistically significant difference*.

### Comparison of location

Both area and length ratios were significantly different between cranial and caudal cervical locations, with smaller ratios in the cranial locations (Table 3).

### Area ratio compared to length ratio

**Figure 2:**
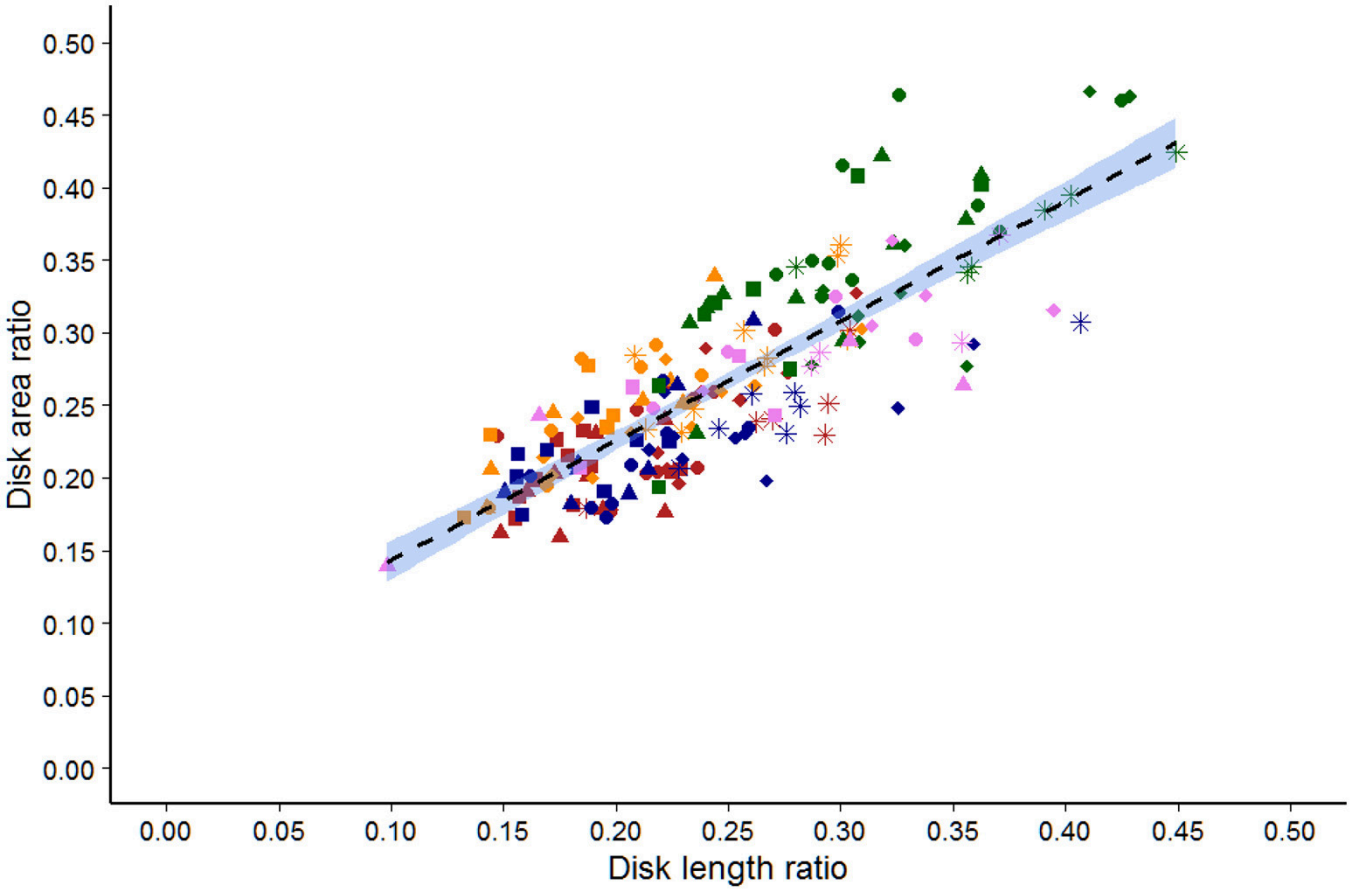
Scatter plot of the area vs. length ratios determined by a single observer for 44 dogs of five breeds. Labrador retriever (Red), French bulldog (orange), Great Dane (blue), Chihuahua (Green), and Dachshund (violet). Squares are C2/C3, triangles are C3/C4, circles are C4/C5, diamonds are C5/C6, and stars are C6/C7 disks.

There was a strong correlation between area and length ratios (rho = 0.821, *p* < 0.001; Figure 2). However, Chihuahuas and French bulldogs tended to have higher area ratios compared to Great Danes and Labrador retrievers that tended to have lower ratios.

### Repeatability

Intraobserver variability for area and length ratio was “excellent” overall (Table 4). When evaluating single breeds and locations, intraobserver variability was “very good” to “excellent,” respectively.

**Table 4:** Repeatability assessed by calculation of the coefficient of variation (CV) for repeated measures concerning disk area and length ratios measured via MRI and stratified based on breed and disk space.

**Measure**	**Variable**	**Level**	**Intra-observer (%)**		**Inter-observer (%)**	
			**Mean**	**Median (IQR)**	**Mean**	**Median (IQR)**
Area ratio	Overall	All	4.59	2.95 (1.46, 5.40)	6.25	6.12 (2.55, 9.50)
	Breed	LR	5.78	3.40 (2.54, 10.96)	8.98	9.89 (4.99, 13.13)
		GD	2.21	1.14 (0.69, 5.02)	3.98	3.45 (2.24, 6.27)
		FB	2.76	1.94 (1.57, 3.23)	3.95	3.98 (1.24, 5.97)
		CH	7.23	5.17 (2.74, 10.56)	7.62	7.41 (5.96, 10.34)
	Disk	C2/C3	7.72	7.79 (2.08, 10.69)	5.28	3.62 (1.43, 9.47)
		C3/4	3.71	1.51 (0.98, 5.40)	6.31	5.95 (2.41, 8.98)
		C4/5	2.74	2.65 (1.44, 3.69)	5.67	3.98 (1.83, 9.83)
		C5/6	3.84	3.65 (1.76, 5.36)	7.01	6.68 (4.61, 9.32)
		C6/7	5.22	2.96 (1.11, 10.11)	6.85	6.32 (3.66, 10.25)
Length ratio	Overall	All	2.77	1.51 (0.88, 4.23)	5.30	5.18 (2.94, 6.62)
	Breed	LR	2.98	2.52 (0.73, 4.35)	4.80	4.34 (2.85, 7.38)
		GD	1.74	1.33 (0.87, 1.92)	4.24	3.44 (1.97, 6.40)
		FB	3.66	3.90 (1.10, 6.30)	6.97	6.47 (3.48, 8.33)
		CH	2.86	1.77 (0.83, 4.25)	5.51	5.54 (3.86, 6.44)
	Disk	C2/C3	4.98	4.96 (1.91, 6.98)	7.73	5.81 (5.27, 9.47)
		C3/4	2.58	1.39 (0.41, 3.29)	5.04	5.66 (3.36, 6.37)
		C4/5	1.88	1.09 (0.84, 3.70)	4.59	4.35 (2.46, 6.61)
		C5/6	1.83	1.42 (0.99, 3.05)	3.76	3.04 (1.84, 5.55)
		C6/7	2.83	1.87 (0.97, 5.28)	5.63	5.22 (3.02, 8.63)

Interobserver variability was “very good” for area and length ratio overall. When evaluating single breeds and locations, interobserver variability was “very good” and “excellent,” respectively.

## Discussion

Various imaging techniques have been used to morphometrically evaluate the canine cervical vertebral column and compare affected and non-affected dogs (19–23). To the authors' knowledge, this is the first study comparing mid-sagittal intervertebral disk to vertebral body area and length ratios of the cervical vertebral column in different dog breeds. Variations in disk to vertebral body ratio may influence local biomechanics and support development of certain degenerative diseases. Validating our hypothesis, results showed that (1) there was a statistically significant difference in area and length ratios between dog breeds and (2) between cranial and caudal cervical locations. Results of this study help to establish reference values of these ratios in various dog breeds and in different cervical locations.

The smallest breed of dog in our study (Chihuahua) had the highest disk to vertebral body area ratio, which was statistically different to all other breeds. A high area ratio means that the size of the vertebral body is relatively small compared to the intervertebral disk. Differences in this ratio could have effects on stresses placed on the intervertebral disk and vertebral structures. It is not possible to draw conclusions from this finding regarding the development of vertebral column diseases. Only 2/10 Chihuahuas were diagnosed with IVDD in contrast to 4/5 Dachshunds, 8/10 French Bulldogs and 5/10 Labradors. Therefore, the clinical significance of a high area ratio is unknown regarding the development of IVDD. Eight of nine Great Danes were diagnosed with OA-CSM; however, their area ratios—while lower compared to Chihuahuas—were not different to Dachshund, French Bulldogs and Labrador Retrievers. Since OA-CSM appears to cause changes mostly to the articular facets, it may be that this disease does not cause morphometric changes of the mid-sagittal vertebral body and disk. It would be interesting to assess these ratios in dogs affected with DA-CSM, such as the Doberman. Unfortunately, insufficient MRIs of the latter breed were available for evaluation in this study, precluding further speculation.

Regardless of overall differences in ratios between dog breeds, all dogs demonstrated smaller disk to vertebral body ratios in cranial cervical locations compared to caudal locations for both area and length. This indicates that cranial vertebrae had larger areas and lengths in relation to their respective disks than caudal vertebrae. Changes in such ratios, with larger and longer cranial vertebral bodies, may influence cervical vertebral column biomechanics. Incidence of canine cervical IVDD is highest in cranial cervical locations (18, 28). The relatively larger and longer vertebral bodies in the cranial cervical vertebral column may lead to increased lever arm stresses on the adjacent disk spaces and may contribute to the higher rate of IVDD in these locations.

In this study, dog breeds were selected to represent chondrodystrophic and non-chondrodystrophic large and small dog breeds. Dog breeds were included if acceptable MRI studies (minimum of 5 animals per breed) were available for the study period. The limited number of dogs in this study (*n* = 44) is similar to other comparable studies (11, 12).

Computed tomography and MRI are common advanced imaging modalities to evaluate the cervical vertebral column. While CT is preferred for assessment of bony structures, MRI is the modality of choice for the evaluation of intervertebral disks and spinal cord (29–31). In our study, area measurements were based on MRI, as we wanted to evaluate the intervertebral disk in addition to vertebral bone. Moreover, MRI is the first choice modality for diagnostic imaging of the head and neck at our institution and therefore provided the highest number of imaging studies per dog breed.

A canine cadaver study, comparing spinal canal and spinal cord measurements on T1 and T2 weighted images on MRI to the actual specimen, showed improved correlation of T2 weighted imaging and anatomical measurements compared to T1 weighted images (12). Consequently in our study, T2 weighted images were used to evaluate vertebrae and disks. Challenges of using T2 weighed images included the inability to clearly identify cortical bone in all areas of the outer vertebral body cortex. For example, the border of the outer cortical bone was not always clear along the dorsal vertebral body bone. In this area, cortical bone was commonly not continuously displayed in the central portion (star in Figure 1A). To address this, evaluators were asked to connect the visible parts of the vertebral cortical boundary with a line of best fit. Another challenge was distinction between the border of the annulus fibrosus and the vertebral endplate cortical bone, as both structures are hypointense in T2 weighted images. The middle distance of the hypointense layer combining the nucleus pulposus and endplate was defined as the border and used as the line of separation for area measurements. While these definitions and solutions helped to standardize the area outline in this study, they may not represent the true outline. Furthermore, it was not always possible to measure at the exact mid-sagittal plane because of the limited number of slices procured. Consequently, the sagittal plane closest to the center was selected for each vertebra and disk.

While patient positioning during MRI was standardized, it is still possible that disk dimensions were influenced by positioning (i.e., effects on intervertebral disk with an extended neck position in a Great Dane with a long vs. a French bulldog with a short cervical vertebral column).

Ideally, disk to vertebral body ratio measurements for reference values should be obtained from entirely normal cervical vertebral columns to exclude possible influence of disease on conformation in adjacent, normal appearing structures. Due to a high incidence of vertebral column disease in the study population, there were insufficient numbers of dogs with entirely normal cervical vertebral columns. Of dogs with clinical or incidental IVDD, measurements were limited to intervertebral disks with a Pfirrmann grade of 2 or less. While the inclusion of disks with mild degenerative changes may not be ideal, area and length measurements are not expected to be influenced. A critique on principle is the inclusion of MRI studies with any grade of disk degeneration. While disks with Pfirrmann grade 3–5 were excluded from analysis, other vertebrae and disks of the same dog were still included. It is unknown if dogs with disk degeneration have different disk to vertebral body ratios in general, even in areas without obvious disease. To determine this, measurements would need to be limited to MRI studies with entirely normal intervertebral disks.

Area measurements of the vertebral bodies and disks were time consuming. To provide a faster and potentially easier method, we compared disk to vertebral area ratios to length ratios. Analysis showed that area to length ratios correlated well-within a particular dog breed but deviated between different breeds. This precludes the use of length ratios when comparing data between different dog breeds. While we attempted to standardize the method for length measurement, we could not account for the inherently variable anatomic shapes of vertebral bodies. It is possible that different methods for determining length ratios may have an improved correlation to area measurements between breeds.

In conclusion, this study provides area and length ratios of intervertebral disks and vertebral bodies in the cervical vertebral column of various dog breeds. These ratios may serve as a baseline for further morphometric evaluation of cervical vertebral column diseases. Whether such ratios can be used to predict the development of specific diseases requires further investigation.

## Author contributions

PD: study design, measurements, and generation of manuscript; CP: study design, interobserver control measurements, and correction of manuscript; GF: statistical analysis; FF: correction of manuscript; BH: study design, interobserver control measurements, and correction of manuscript.

### Conflict of interest statement

The authors declare that the research was conducted in the absence of any commercial or financial relationships that could be construed as a potential conflict of interest.

## Abbreviations

CT: computed tomography
IVDD: intervertebral disk disease
IVD: intervertebral disk
MRI: magnetic resonance imaging
CSM: cervical spondylomyelpathy
OA-CSM: osseous-associated cervical spondylomyelpathy
DA-CSM: disk-associated cervical spondylomyelpathy
LR: Labrador retriever
GD: Great Dane
FB: French bulldog
CH: Chihuahua
DH: Dachshund

## References

[1] Utkualp N Ercan I. Anthropometric measurements usage in medical sciences. Biomed Res Int. (2015) 2015:404261. 10.1155/2015/404261 26413519 PMC4564618

[2] Shiraishi N Katayama A Nakashima T Yamada S Uwabe C Kose K. Morphology and morphometry of the human embryonic brain: a three-dimensional analysis. Neuroimage (2015) 115:96–103. 10.1016/j.neuroimage.2015.04.044 25934469

[3] Vrancken AC Crijns SP Ploegmakers MJ O'Kane C van Tienen TG Janssen D. 3D geometry analysis of the medial meniscus–a statistical shape modeling approach. J Anat. (2014) 225:395–402. 10.1111/joa.12223 25052030 PMC4174023

[4] Pazzaglia UE Donzelli CM Izzi C Baldi M Di Gaetano G Bondioni M. Thanatophoric dysplasia. Correlation among bone X-ray morphometry, histopathology, and gene analysis. Skeletal Radiol. (2014) 43:1205–15. 10.1007/s00256-014-1899-1 24859745

[5] Dabanoglu I. Normal morphometry of the thoracic aorta in the German shepherd dog: a computed tomographic study. Anat Histol Embryol. (2007) 36:163–7. 10.1111/j.1439-0264.2006.00717.x 17535345

[6] Ondreka N Amort KH Stock KF Tellhelm B Klumpp SW Kramer M. Skeletal morphology and morphometry of the lumbosacral junction in German shepherd dogs and an evaluation of the possible genetic basis for radiographic findings. Vet J. (2013) 196:64–70. 10.1016/j.tvjl.2012.07.015 22921082

[7] Breit S Künzel W. Osteological features in pure-bred dogs predisposing to cervical spinal cord compression. J Anat. (2001) 199(Pt 5):527–37. 10.1046/j.1469-7580.2001.19950527.x 11760884 PMC1468364

[8] Breit S Künzel W. A morphometric investigation on breed-specific features affecting sagittal rotational and lateral bending mobility in the canine cervical spine (C3–C7). Anat Histol Embryol. (2004) 33:244–50. 10.1111/j.1439-0264.2004.00546.x 15239817

[9] Drost WT Lehenbauer TW Reeves J. Mensuration of cervical vertebral ratios in Doberman pinschers and Great Danes. Vet Radiol Ultrasound (2002) 43:124–31. 10.1111/j.1740-8261.2002.tb01659.x 11954807

[10] Fourie SL Kirberger RM. Relationship of cervical spinal cord diameter to vertebral dimensions: a radiographic study of normal dogs. Vet Radiol Ultrasound (1998) 40:137–43. 10225524 10.1111/j.1740-8261.1999.tb01898.x

[11] Seo E Choi J Choi M Yoon J. Computed tomographic evaluation of cervical vertebral canal and spinal cord morphometry in normal dogs. J Vet Sci. (2014) 15:187. 10.4142/jvs.2014.15.2.187 24136210 PMC4087219

[12] Hecht S Huerta MM Reed RB. Magnetic resonance imaging (MRI) spinal cord and canal measurements in normal dogs. Anat Histol Embryol. (2014) 43:36–41. 10.1111/ahe.12045 23488993 PMC3933761

[13] Murthy VD Gaitero L Monteith G. Clinical and magnetic resonance imaging (MRI) findings in 26 dogs with canine osseous-associated cervical spondylomyelopathy. Can Vet J. (2014) 55:169–74. 24489397 PMC3894878

[14] Toombs JP Cervical intervertebral disc disease in dogs. Compend Contin Educ Pract Vet. (1992) 14:1477–86.

[15] Hansen HJ A pathologic-anatomical study on disk degeneration in the dog. Acta Orthop Scand. (1952) 11:1–117. 10.3109/ort.1952.23.suppl-11.01 14923291

[16] Olsson SE Hansen HJ. Cervical disc protrusions in the dog. J Am Vet Med Assoc. (1952) 121:361–70. 13022507

[17] da Costa RC Cervical spondylomyelopathy (Wobbler syndrome) in dogs. Vet Clin Small Anim Pract. (2010) 40:881–913. 10.1016/j.cvsm.2010.06.003 20732597

[18] Hakozaki T Iwata M Kanno N Harada Y Yogo T Tagawa M. Cervical intervertebral disk herniation in chondrodystrophoid and nonchondrodystrophoid small-breed dogs: 187 cases (1993–2013). J Am Vet Med Assoc. (2015) 247:1408–11. 10.2460/javma.247.12.1408 26642135

[19] Decker SD Gielen IM Duchateau L van Bree HJ Waelbers T Bavegems V. Morphometric dimensions of the caudal cervical vertebral column in clinically normal Doberman Pinschers, English Foxhounds and Doberman Pinschers with clinical signs of disk-associated cervical spondylomyelopathy. Vet J. (2012) 191:52–7. 10.1016/j.tvjl.2010.12.017 21257325

[20] Decker SD Gielen IM Duchateau L Saunders JH van Bree HJ Polis I. Magnetic resonance imaging vertebral canal and body ratios in Doberman Pinschers with and without disk-associated cervical spondylomyelopathy and clinically normal English Foxhounds. Am J Vet Res. (2011) 72:1496–504. 10.2460/ajvr.72.11.1496 22023128

[21] Martin-Vaquero P da Costa RC Lima CG. Cervical spondylomyelopathy in Great Danes: a magnetic resonance imaging morphometric study. Vet J. (2014) 201:64–71. 10.1016/j.tvjl.2014.04.011 24888675 PMC4169205

[22] Decker SD Gielen IM Duchateau L Volk HA Van Ham LM. Intervertebral disk width in dogs with and without clinical signs of disk associated cervical spondylomyelopathy. BMC Vet Res. (2012) 8:126. 10.1186/1746-6148-8-126 22839697 PMC3411421

[23] Costa RC Parent JM Dobson H Dobson H Holmberg DL Lamarre J. Morphologic and morphometric magnetic resonance imaging features of Doberman Pinschers with and without clinical signs of cervical spondylomyelopathy. Am J Vet Res. (2006) 67:1601–12. 10.2460/ajvr.67.9.1601 16948609

[24] Lim J Yoon Y Hwang T Lee HC. Novel vertebral computed tomography indices in normal and spinal disorder dogs. J Vet Sci. (2018) 19:296–300. 10.4142/jvs.2018.19.2.296 29169229 PMC5879078

[25] Bonelli MA da Costa RC Martin-Vaquero P Lima CG. Comparison of angle, shape, and position of articular processes in Dobermans and Great Danes with and without cervical spondylomyelopathy. BMC Vet Res. (2017) 13:77. 10.1186/s12917-017-0997-4 28340590 PMC5366139

[26] Laing AC Cox R Tetzlaff W Oxland T. Effects of advanced age on the morphometry and degenerative state of the cervical spine in a rat model. Anat Rec. (2011) 294:1326–36. 10.1002/ar.21436 21714115

[27] Bergknut N Auriemma E Wijsman S Voorhout G Hagman R Lagerstedt AS. Evaluation of intervertebral disk degeneration in chondrodystrophic and nonchondrodystrophic dogs by use of Pfirrmann grading of images obtained with low-field magnetic resonance imaging. Am J Vet Res. (2011) 72:893–8. 10.2460/ajvr.72.7.893 21728849

[28] Cherrone KL Dewey CW Coates JR Bergman RL. A retrospective comparison of cervical intervertebral disk disease in nonchondrodystrophic large dogs versus small dogs. J Am Anim Hosp Assoc. (2004) 40:316–20. 10.5326/0400316 15238562

[29] Decker SD Gielen IM Duchateau L Polis I Van Bree HJ Van Ham LM. Agreement and repeatability of linear vertebral body and canal measurements using computed tomography (CT) and low field magnetic resonance imaging (MRI). Vet Surg. (2010) 39:28–34. 10.1111/j.1532-950X.2009.00559.x 20210941

[30] Gopal MS Jeffery ND. Magnetic resonance imaging in the diagnosis and treatment of a canine spinal cord injury. J Small Anim Pract. (2001) 42:29–31. 10.1111/j.1748-5827.2001.tb01981.x 11219821

[31] Levitski RE Lipsitz D Chauvet AE. Magnetic resonance imaging of the cervical spine in 27 dogs. Radiol Ultrasound (1999) 40:332–41. 10463823 10.1111/j.1740-8261.1999.tb02120.x

